# OA18 Beyond sicca: catastrophic neurological primary Sjögren’s syndrome in a male patient with severe neuropathy, recurrent cerebral infarction, and critical limb ischaemia

**DOI:** 10.1093/rap/rkag113.018

**Published:** 2026-11-03

**Authors:** Asma Qazi, Anupama Nandagudi

**Affiliations:** Rheumatology Department, Basildon University Hospital, Basildon, United Kingdom; Rheumatology Department, Basildon University Hospital, Basildon, United Kingdom

## Abstract

**Introduction:**

Primary Sjogren’s syndrome (pSS) is a chronic autoimmune disorder classically characterised by exocrinopathy; however, extraglandular manifestations may dominate the clinical presentation and contribute substantially to morbidity. Neurological involvement is relatively uncommon, with peripheral neuropathy affecting approximately 15% of patients and central nervous system vasculitis occurring rarely. Male patients represent a particularly under-recognised subgroup, as pSS predominantly affects women. We report a six-year longitudinal case of a male patient with pSS diagnosed in the absence of sicca symptoms, who developed severe axonal sensorimotor polyneuropathy, recurrent cerebral vasculitic infarctions, and critical upper-limb ischaemia with livedo reticularis, representing an exceptionally rare clinical phenotype.

**Case description:**

A 52-year-old man was referred to the rheumatology service with a 12-week history of progressive bilateral foot and ankle pain associated with worsening lower-limb weakness. Initial investigations demonstrated significant immunological abnormalities despite the absence of classical sicca symptoms. He denied both dry eyes and dry mouth; however, Schirmer’s testing was strongly positive bilaterally. Serological evaluation revealed rheumatoid factor >600 IU/mL, positive ANA (6.9), anti-Ro (SSA) and anti-La (SSB) antibodies, hypergammaglobulinaemia on serum protein electrophoresis, and isolated C4 hypocomplementaemia with normal C3 levels, suggestive of immune complex-mediated disease with classical complement pathway activation. Anti-dsDNA antibodies, ANCA, paraneoplastic markers, and infective screens were negative. Nerve conduction studies demonstrated an acute axonal sensorimotor polyneuropathy affecting the lower limbs, leading to a diagnosis of primary Sjögren’s syndrome (pSS) with neurological involvement.

Over subsequent months, neurological deficits progressed rapidly to complete loss of weight-bearing capacity, resulting in wheelchair dependence and profound functional impairment. Treatment with six cycles of intravenous cyclophosphamide and corticosteroids achieved partial neurological recovery, with improvement in motor function and electrophysiological parameters. Maintenance azathioprine was poorly tolerated because of severe gastrointestinal adverse effects and was replaced with mycophenolate mofetil. Although disease control was achieved, the clinical course was complicated by steroid-induced talar osteonecrosis, pericarditis, and recurrent opportunistic infections.

Two years after diagnosis, following a period of treatment non-adherence, the patient developed right upper-limb weakness, dysarthria, and left facial palsy. MRI brain demonstrated a left frontoparietal cortical infarction considered most consistent with cerebral vasculitis related to pSS. Concurrently, he developed extensive livedo reticularis, limb discolouration, and critical right upper-limb ischaemia despite preserved arterial patency on CT angiography, supporting a small-vessel vasculitic process. Further cyclophosphamide, rituximab, and anticoagulation resulted in substantial recovery. At six-year follow-up, he remained clinically stable on mycophenolate mofetil monotherapy with no further neurological progression.

**Discussion:**

This case highlights an exceptionally severe extraglandular neurological phenotype of primary Sjögren’s syndrome, characterised by progressive axonal sensorimotor polyneuropathy, recurrent cerebral ischaemic infarction attributed to vasculitis, and critical upper-limb small-vessel ischaemia. While pSS is classically recognised as an autoimmune exocrinopathy, neurological involvement is increasingly acknowledged as a major determinant of morbidity. Peripheral neuropathy affects approximately 15% of patients and may precede the diagnosis of pSS. The present case reinforces this observation, as the patient’s initial manifestation was a rapidly progressive axonal neuropathy occurring in the complete absence of sicca symptoms. Such presentations highlight the importance of considering pSS in the differential diagnosis of otherwise unexplained neuropathy and is consistent with the 2016 ACR/EULAR criteria, which permit diagnosis on objective serological and glandular findings alone, irrespective of sicca symptoms.

A striking feature throughout the disease course was persistent isolated C4 hypocomplementaemia. This biomarker has been consistently associated with systemic disease activity and adverse outcomes in pSS, including peripheral neuropathy, cutaneous vasculitis, cryoglobulinaemia, lymphoma development, and reduced survival. The persistently depressed C4 levels paralleled major clinical events, including neurological progression, cerebral infarction, and peripheral vascular ischaemia, suggesting ongoing immune complex-mediated vascular injury. Serial complement monitoring thus provided a valuable disease activity indicator, informing decisions to escalate immunosuppression treatment.

The cerebrovascular manifestations observed here are particularly noteworthy. Cerebral vasculitis is an exceptionally rare complication of pSS, with only a limited number of cases reported in the literature. Recurrent cortical infarction alongside severe peripheral neuropathy and upper-limb vasculitic ischaemia represents an unreported clinical constellation. Management required aggressive sequential immunosuppression with cyclophosphamide induction, mycophenolate mofetil maintenance, and rituximab for refractory disease, resulting in significant functional recovery and long-term stabilisation. This case emphasises the need for heightened clinical awareness of severe neurological pSS and supports early, sustained immunosuppressive intervention in patients demonstrating high-risk vasculitic features.

**Key learning points:**

**1: Primary Sjögren’s Syndrome can occur without sicca** pSS should not be regarded solely as an exocrine gland disease. This case demonstrates that catastrophic systemic manifestations can occur without sicca symptoms. Despite denying sicca throughout six years, diagnostic criteria were fulfilled through objective Schirmer’s testing and characteristic serology. Subjective dryness is not essential for diagnosis. Clinicians must pursue objective glandular assessment whenever unexplained neurological manifestations coexist with supportive immunological findings.

**2: Unexplained Axonal Neuropathy Warrants Screening for pSS** Peripheral neuropathy may be the presenting manifestation of pSS and can precede diagnosis by several years. In unexplained axonal sensorimotor polyneuropathy, comprehensive autoimmune screening is essential. Strongly positive RF, ANA, anti-Ro/La antibodies, hypergammaglobulinaemia, and isolated C4 hypocomplementaemia constitute a highly characteristic immunological profile. Early diagnosis and prompt immunosuppression may prevent irreversible axonal loss and loss of functional independence.

**3: Persistent C4 Hypocomplementaemia Identifies High-Risk Disease** Isolated C4 hypocomplementaemia was a consistent feature throughout this patient’s course and served as an important disease activity biomarker. Low C4 in pSS is associated with vasculitic neuropathy, cutaneous vasculitis, cryoglobulinaemia, lymphoma, and reduced survival. Serial complement monitoring provided a valuable longitudinal marker of immune complex-mediated disease activity, guiding immunosuppressive escalation during periods of clinical deterioration.

**4: Cerebral Vasculitis in pSS is Rare but devastating** The combination of severe peripheral neuropathy, cerebral vasculitic infarction, and upper-limb small-vessel ischaemia represents an exceptionally rare pSS phenotype. New focal neurological deficits in established or suspected pSS should prompt urgent neuroimaging and multidisciplinary review. Early recognition of cerebral vasculitis is critical, given its association with recurrent and potentially irreversible neurological injury.

**5: Multidisciplinary Management Is Essential** Complex pSS with neurological and vascular involvement requires coordinated long-term management across rheumatology, neurology, stroke medicine, dermatology, vascular surgery, and rehabilitation services to establishing diagnosis, monitoring disease activity, and achieving durable clinical stability.

